# A bibliographic database on economic analysis of natural forest disturbances

**DOI:** 10.1016/j.dib.2018.08.128

**Published:** 2018-08-31

**Authors:** Claire Montagné-Huck, Marielle Brunette

**Affiliations:** Université de Lorraine, Université de Strasbourg, AgroParisTech, CNRS, INRA, BETA, 54000, Nancy, France

## Abstract

The data presented here are related to the research paper entitled “Economic Analysis of Natural Forest Disturbances: A Century of Research” (Montagné-Huck and Brunette, 2018). Natural disturbances have always affected forest ecosystems, altering or disrupting the flows of goods (wood, non-timber products, etc.) and services (scenic and recreation values, leisure pursuits, clean water, regulation of floods, etc.) provided by forests to human societies. Economic analysis can help private or public decision-makers take forest policy decisions by understanding the causes and consequences of forest disturbances, as well as evaluating tradeoffs in alternative policy scenarios. In consequence, the economic literature about natural disturbances is very rich and diversified.

This paper describes a bibliographic database gathering some 340 scientific papers related to the economic analysis of forest natural disturbances. Papers have been inventoried primarily thanks to searches on databases (JSTOR, ScienceDirect, Ingentaconnect and NRC Research Press) using specific keywords and the dataset have been completed by searching through literature-cited sections of papers. Relevant papers have been manually encoded into a database (Excel file) taking into consideration each type of hazard (storm, fire, snow, pests and diseases, etc.) and different economic approaches. Data cover papers published in English from 1916 to 2014.

**Specifications Table**

**Table:** 

Subject area	Economics
More specific subject area	Economic analysis of natural forest disturbances
Type of data	Table
How data was acquired	Bibliographic search and manual encoding from research articles
Data format	Raw
Experimental factors	340 articles dealing with economic analysis of natural disturbances (wildfire, pest, pathogen, storm, wildlife, ice/snow and other or multiple hazards) were obtained through an extensive literature review process and were included according to the inclusion criteria stated in [1]. Data concerning publication references, case study specification and method used in the paper were extracted from the included studies and described below.
Experimental features	Literature review was performed, analyzing the 340 articles included according to each hazard type.
Data source location	Africa, America, Asia, Europe, Oceania
Data accessibility	Data provided with this article

**Value of the data**

• Data cover 340 scientific articles in the field of economic analysis of forest disturbances.
• Data allow for literature reviews in the field of economic analysis of forest disturbances either by hazard or by economic approach.
• Data were used in [1] in order to conduct a survey on how economic analysis deals with forest disturbances related issues, synthesizing the existing knowledge, characterizing forest disturbances and identifying gaps in the literature.

## 1 Data

The database is an original Excel file that includes some 340 scientific papers dealing with the economic analysis of forest natural disturbances. Data cover papers published in English from 1916 to 2014. Each line of the sheet represents an article; each column of the sheet represents a variable. Papers are encoded in the database according to 3 types of variables: variables concerning the references of the article; variables specifying the case study, if any; variables providing information on the method used by the author(s). Table 1 provides the description and encoding values for the variables (note that detailed variable description and encoding are also available in the spreadsheet –cf. “read-me” sheet).

**Table 1:** Variables registered in the database (adapted from [1]).

Type of variable	Variable name	Content	Format	Comments	Descriptive data
Reference of the paper	Id-Art	Identifier of the article	Character	Number (n) corresponds to the paper number and letter (l) corresponds to hazard׳s first letters (w=wildfire, pe=pest, pa=pathogen, s=storm, wd=wildlife damage, is=ice snow, mh=multiple hazards)	*N* = 340
		nl			
	Hazard	Type of hazard studied in the paper	Character	wildfire	n.w = 210
				pest	n.pe = 65
				pathogen	n.pa = 16
				storm	n.s = 16
				wildlife damage	n.wd = 5
				ice snow	n.is = 4
				multiple hazards	n.mh = 24
	Author 1	Name of the first author of the paper	Character		
	Country A1	Country of the first author of the paper	Character		Africa = 0
					America = 271
					Asia = 7
					Europe = 51
					Oceania = 10
	Year	Year of publication	Numeric		Min = 1916
					Max = 2014
	Art tit	Article title	Character		
	Review	Journal title	Character		
Case study specification	Country CS	Country where the case study takes place	Character		Africa = 2
					America = 274
					Asia = 4
					Europe = 50
					Oceania = 6
					Worldwide = 1
	State CS	State of the country where the case study takes place	Character		
	Forest type	Tree species	Character	Scientific names	
Method used in the paper	Approach	Theoretical/Empirical/Both	Character		
	Category	Valuing economic impact/Decision making	Character	The meaning of the variable “category” is defined as follows:	n.impact = 79
					n.response = 261
				“Valuing the economic impacts of forest disturbances” includes the articles in which an accounting of the costs and economic losses due to forest disturbances is carried out (e.g., the impact of forest disturbances on timber markets, willingness-to-pay for programs to reduce the risk, etc.).	
				“Decision-making in response to forest disturbance” covers articles that serve as a support to decision-makers (e.g., insurance decision, investment decision, optimal rotation under risk, etc.).	
	Type of analysis	Type of economic approach	Character	Cost and benefit assessment (CBA)	n.CBA = 89
				Efficiency analysis (EA)	n.EA = 127
				Risk management (RM)	n.RM = 53
				Wildland-urban interface (WUI)	n.WUI = 70
				Literature review (LR)	n.LR = 1
	Kwd Method	Keywords relative to the method used	Character		
	Kwd Topic	Keywords relative to the topic analyzed	Character		
	Pre-event	Study’s time positioning relative to hazardous event occurrence	Dummy	1 = yes, 0 = no	n.pre-event = 126
	Event		Dummy		n.event = 97
	Post-event		Dummy		n.post-event = 154
	Temporality	Static or dynamic analysis	Character	Static/Dynamic	n.static = 150
					n.dynamic = 190
	Spatial analysis	Spatial analysis or not	Character	Yes/No	n.yes = 66
					n.no = 274
	Purpose of the article	Free text describing the aim of the paper	Character		
	Abstract	Abstract of the paper if any, main conclusions otherwise	Character		
	Reference	Comprehensive article reference	Character		

## 2 Experimental design, materials and methods

We conducted a systematic literature research during the spring of 2014 using combinations of three keywords on four databases/search engines: JSTOR, ScienceDirect, Ingentaconnect and NRC Research Press. Each search combined the keywords for searching in papers’ full-text as follows: Forest* AND Economic* AND specific keyword related to the analyzed disturbance. For each hazard, we tried the following specific keywords:

• Wildfire: wildfire OR fire OR prescribed burning OR fuel management OR fuel treatment OR fuel reduction OR fuel break
• Pest: pest OR epidemic OR infestation OR insect
• Pathogen: pathogen OR disease OR infection
• Storm: storm OR hurricane OR tornado OR cyclone OR typhoon OR wind OR windstorm OR windthrow OR gale OR straight line wind
• Wildlife damage: browsing OR debark
• Snow – Ice: snow OR ice
• Other abiotic disturbances: drought OR flood OR landslide OR volcano
• Other, multiple hazards: catastrophe OR damage OR mortality OR disturbance OR hazard OR risk OR stochastic OR uncertainty.

Complementarily, we used the reference lists of the identified papers to add other relevant articles into the database. We only include articles that are published in English. We then collected 340 papers. The comprehensive list is presented in Appendix A: Supplementary material.

Note that we did not find any article using the defined keywords for the category “Other abiotic disturbances”. Thus, such disturbances (drought, flood, landslide, and volcano) do not appear in the database.

“Other, multiple hazards” category gathers papers that we cannot classify into the previous ones because they deal with non-specified natural hazard or with several natural hazards or with another type of hazard (e.g., hydrogeological hazard). See [1] for details.
