# Supplementary material for: A bibliographic database on economic analysis of natural forest disturbances
Source: Data Brief. 2018 Aug 31;20:662–6. doi: 10.1016/j.dib.2018.08.128 (PMC6129737; doi:10.1016/j.dib.2018.08.128)
Supplement: Supplementary file 1 — Supplementary material [file mmc1.docx]

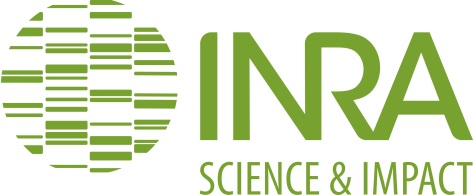


The authors, Claire Montagné-Huck and Marielle Brunette ensure that there is no financial/personal interest or belief that could affect their objectivity.

We state explicitly that no conflict of interest exists.

Best regards,

Claire Montagné-Huck, Brunette Marielle
