# Supplementary material for: A bibliographic database on economic analysis of natural forest disturbances
Source: Data Brief. 2018 Aug 31;20:662–6. doi: 10.1016/j.dib.2018.08.128 (PMC6129737; doi:10.1016/j.dib.2018.08.128)
Supplement: Supplementary file 2 — Supplementary material [file mmc2.docx]

**Appendix A: List of references included in the database Montagné-Huck and Brunette^[[1]](#footnote-1)^ (by hazard and alphabetic order)**

**Wildfires** n_1_=210.

[187-w] Abbas, D., Arnosti, D., 2013. Economics and Logistics of Biomass Utilization in the Superior National Forest. J. Sustain. Forest. 32(1), 41–57.

[170-w] Abbas, D., Current, D., Ryans, M., Taff, S., Hoganson, H., Brooks, K.N., 2011. Harvesting forest biomass for energy – An alternative to conventional fuel treatments: Trials in the Superior National Forest, USA. Biomass Bioenerg. 35(11), 4557–4564.

[127-w] Abt, K.L., Prestemon, J.P., 2006. Timber markets and fuel treatments in the western US. Nat. Resour. Model. 19(1), 15–43.

[120-w] Acuna, M.A., Palma, C.D., Cui, W., Martell, D.L., Weintraub, A., 2010. Integrated spatial fire and forest management planning. Can. J. Forest Res. 40(12), 2370–2383.

[113-w] Adams, D.M., Latta, G.S., 2004. Effects of a Forest Health Thinning Program on Land and Timber Values in Eastern Oregon. J. Forest. 102(8), 9–13.

[150-w] Amacher, G.S., Malik, A.S., Haight, R.G., 2005. Forest landowner decisions and the value of information under fire risk. Can. J. Forest Res. 35(11), 2603–2615.

[8-w] Amacher, G.S., Malik, A.S., Haight, R.G., 2005. Not Getting Burned: The Importance of Fire Prevention in Forest Management. Land Econ. 81(2), 284–302.

[84-w] Amacher, G.S., Malik, A.S., Haight, R.G., 2005. Non-industrial private landowners, fires, and the wildland–urban interface. For. Policy Econ. 7(5), 796–805.

[70-w] Amacher, G.S., Malik, A.S., Haight, R.G., 2006. Reducing Social Losses from Forest Fires. Land Econ. 82(3), 367–383.

[164-w] Barreal, J., Loureiro, M.L., Picos, J., 2014. On insurance as a tool for securing forest restoration after wildfires. For. Policy Econ. 42, 15–23.

[124-w] Baumgartner, D.C., 1987. Salvaging Fire-Damaged Timber in Michigan. North. J. Appl. For. 4(3), 149–152.

[141-w] Becker, D.R., Larson, D., Lowell, E.C., 2009. Financial considerations of policy options to enhance biomass utilization for reducing wildfire hazards. For. Policy Econ. 11(8), 628–635.

[167-w] Becker, D.R., Nechodom, M., Barnett, A., Mason, T., Lowell, E.C., Shelly, J., Graham, D., 2009. Assessing the role of federal community assistance programs to develop biomass utilization capacity in the western United States. For. Policy Econ. 11(2), 141–148.

[34-w] Beichler, W.K., 1940. Fire Control Objectives and Public Finance. J. Forest. 38(4), 333–338.

[28-w] Bellinger, M.D., Kaiser, H.F., Harrison, H.A., 1983. Economic Efficiency of Fire Management on Nonfederal Forest and Range Lands. J. Forest. 81(6), 373–378.

[106-w] Bennetton, J., Cashin, P., Jones, D., Soligo, J., 1998. An economic evaluation of bushfire prevention and suppression. Aust. J. Agr. Resour. Ec. 42(2), 149–175.

[205-w] Berry, A.H., Donovan, G., Hesseln, H., 2006. Prescribed Burning Costs and the WUI: Economic Effects in the Pacific Northwest. West. J. Appl. For. 21(2), 72–78.

[21-w] Berry, A.H., Hesseln, H., 2004. The Effect of the Wildland-Urban Interface on Prescribed Burning Costs in the Pacific Northwestern United States. J. Forest. 102(6), 33–37.

[66-w] Boxall, P.C., Englin, J.E., 2008. Fire and Recreation Values in Fire-Prone Forests: Exploring an Intertemporal Amenity Function Using Pooled RP-SP Data. J. Agr. Resour. Econ. 33(1), 19–33.

[147-w] Boxall, P.C., Watson, D.O., Englin, J., 1996. Backcountry recreationists’ valuation of forest and park management features in wilderness parks of the western Canadian Shield. Can. J. Forest Res. 26(6), 982–990.

[51-w] Boychuk, D., Martell, D.L., 1996. A Multistage Stochastic Programming Model for Sustainable Forest-Level Timber Supply Under Risk of Fire. Forest Sci. 42(1), 10–26.

[114-w] Brian, P., John, N., 2005. Estimating harvest schedules and profitability under the risk of fire disturbance. Can. J. Forest Res. 35(6), 1378–1388.

[121-w] Brown, B.G., Murphy, A.H., 1988. On the economic value of weather forecasts in wildfire suppression mobilization decisions. Can. J. Forest Res. 18(12), 1641–1649.

[160-w] Brown, R.N.K., Rosenberger, R.S., Kline, J.D., Hall, T.E., Needham, M.D., 2008. Visitor Preferences for Managing Wilderness Recreation after Wildfire. J. Forest. 106(1), 9–16.

[53-w] Brown, T.C., Boster, R.S., 1978. On the Economics of Timber Damage Appraisal for Public Forests. J. Forest. 76(12), 777–780.

[176-w] Burtner, C.E., 1966. The Economics of a Fire protection program. Fire Technol. 2(1), 5p.

[177-w] Busby, G., Albers, H.J., 2010. Wildfire Risk Management on a Landscape with Public and Private Ownership: Who Pays for Protection? Environ. Manage. 45, 296-310.

[59-w] Busby, G.M., Albers, H.J., Montgomery, C.A., 2012. Wildfire Risk Management in a Landscape with Fragmented Ownership and Spatial Interactions. Land Econ. 88(3), 496–517.

[5-w] Butry, D.T., Mercer, D.E., Prestemon, J.P., Pye, J.M., Holmes, T.P., 2001. What Is the Price of Catastrophic Wildfire? J. Forest. 99(11), 9–17.

[16-w] Calkin, D., Gebert, K., 2006. Modeling Fuel Treatment Costs on Forest Service Lands in the Western United States. West. J. Appl. For. 21(4), 217–221.

[117-w] Calkin, D.E., Gebert, K.M., Jones, G.J., Neilson, R.P., 2005. Forest Service Large Fire Area Burned and Suppression Expenditure Trends, 1970–2002. J. Forest. 103(4), 179–183.

[101-w] Calkin, D.E., Thompson, M.P., Finney, M.A., Hyde, K.D., 2011. A Real-Time Risk Assessment Tool Supporting Wildland Fire Decision making. J. Forest. 109(5), 274–280.

[71-w] Cohan, D., Haas, S.M., Radloff, D.L., Yancik, R.F., 1984. Using Fire in Forest Management: Decision Making under Uncertainty. Interfaces. 14(5), 8–19.

[55-w] Cohan, D., Haas, S., Roussopoulos, P.J., 1983. Decision Analysis of Silvicultural Prescriptions and Fuel Management Practices on an Intensively Managed Commercial Forest. Forest Sci. 29(4), 858–870.

[195-w] Collins, T.W., 2005. Households, forests, and fire hazard vulnerability in the American West: A case study of a California community. Env. Hazards. 6(1), 23–37.

[79-w] Couture, S., Reynaud, A., 2011. Forest management under fire risk when forest carbon sequestration has value. Ecol. Econ. 70(11), 2002–2011.

[25-w] Coyle, L., 1929. A Basis for Determining Proper Expenditures for Fire Protection. J. Forest. 27(2), 148–150.

[104-w] Crowley, C.S.L., Malik, A.S., Amacher, G.S., Haight, R.G., 2009. Adjacency Externalities and Forest Fire Prevention. Land Econ. 85(1), 162–185.

[47-w] Dale, L., Aplet, G., Wilmer, B., 2005. Wildland Fire Use and Cost Containment: A Colorado Case Study. J. Forest. 103(6), 314–318.

[211-w] Davis, E.J., Moseley, C., Nielsen-Pincus, M., Jakes, P.J., 2014. The Community Economic Impacts of Large Wildfires: A Case Study from Trinity County, California. Soc. Natur. Resour. 27(9), 983–993.

[203-w] DeLasaux, M.J., Hartsough, B.R., Raffaele, S., Natascia, M., 2009. Small Parcel Fuel Reduction with a Low-Investment, High-Mobility Operation. West. J. Appl. For. 24(4), 205–213.

[148-w] Demchik, M.C., Abbas, D., Current, D., Arnosti, D., Theimer, M., Johnson P., 2009. Combining Biomass Harvest and Forest Fuel Reduction in the Superior National Forest, Minnesota. J. Forest. 107(5), 235–241.

[19-w] Donovan, G.H., 2005. A Comparison of the Costs of Forest Service and Contract Fire Crews in the Pacific Northwest. West. J. Appl. For. 20(4), 233–239.

[105-w] Donovan, G.H., Brown, T.C., 2005. An Alternative Incentive Structure for Wildfire Management on National Forest Land. Forest Sci. 51(5), 387–395.

[131-w] Donovan, G.H., Champ, P.A., Butry, D.T., 2007. Wildfire Risk and Housing Prices: A Case Study from Colorado Springs. Land Econ. 83(2), 217–233.

[2-w] Donovan, G.H., Rideout, D.B., 2003. A Reformulation of the Cost Plus Net Value Change (C+NVC) Model of Wildfire Economics. Forest Sci. 49(2), 318–323.

[3-w] Donovan, G.H., Rideout, D.B., 2003. An Integer Programming Model to Optimize Resource Allocation for Wildfire Containment. Forest Sci. 49(2), 31-335.

[175-w] Donovan, G.H., Noordijk, P., 2005. Assessing the Accuracy of Wildland Fire Situation Analysis (WFSA) Fire Size and Suppression Cost Estimates. J. Forest. 103(1), 10–13.

[13-w] Englin, J., Boxall, P.C., Chakraborty, K., Watson, D.O., 1996. Valuing the Impacts of Forest Fires on Backcountry Forest Recreation. Forest Sci. 42(4), 450–455.

[7-w] Englin, J., Boxall, P., Hauer, G., 2000. An Empirical Examination of Optimal Rotations in a Multiple-Use Forest in the Presence of Fire Risk. J. Agr. Resour. Econ. 25(1), 14-27.

[41-w] Englin, J., Loomis, J., González-Cabán, A., 2001. The dynamic path of recreational values following a forest fire: a comparative analysis of states in the Intermountain West. Can. J. Forest Res. 31(10), 1837–1844.

[74-w] Espelta, J.M., Retana, J., Habrouk, A., 2003. An economic and ecological multicriteria evaluation of reforestation methods to recover burned Pinus nigra forests in NE Spain. Forest Ecol. Manag. 180(1–3), 185–198.

[178-w] Fanariotu, I., Skuras, D., 2004. The Contribution of scenic beauty indicators in estimating environmental welfare measures: a case study. Soc. Indic. Res. 65, 145-165.

[179-w] Ferreira, L., Constantino, M., Borges, J.G., 2014. A stochastic approach to optimize Maritime pine (Pinus pinaster Ait.) stand management scheduling under fire risk. An application in Portugal. Ann. Oper. Res. 219, 359-377.

[40-w] Flint, H.H., 1924. The Appraisal of Forest Fire Damages. J. Forest. 22(2), 154–161.

[24-w] Flint, H.R., 1928. Adequate Fire Control. J. Forest. 26(5), 624–638.

[60-w] Fried, J.S., Winter, G.J., Gilless, J.K., 1999. Assessing the benefits of reducing fire risk in the Wildland-urban interface: a contingent valuation approach. Int. J. Wildland Fire. 9(1), 9–20.

[137-w] Gautam, S., Pulkki, R., Shahi, C., Leitch, M., 2010. Economic and energy efficiency of salvaging biomass from wildfire burnt areas for bioenergy production in northwestern Ontario: A case study. Biomass Bioenerg. 34(11), 1562–1572.

[112-w] Gebert, K.M., Black, A.E., 2012. Effect of Suppression Strategies on Federal Wildland Fire Expenditures. J. Forest. 110(2), 65–73.

[44-w] Grah, R.F., Long, A., 1971. Three California Fuelbreaks: Costs and Benefits. J. Forest. 69(2), 89–93.

[123-w] González, J.R., Pukkala, T., Palahí, M., 2005. Optimising the management of Pinus sylvestris L. stand under risk of fire in Catalonia (north-east of Spain). Ann. For. Sci. 62(6), 493–501.

[196-w] González-Cabán, A., 1993. Improving the U.S. Forest Service's Fire Management Planning System Capabilities to Use Non-Market Values. Wildfire. 1(1), 16-21.

[18-w] González-Cabán, A., 1997. Managerial and Institutional Factors Affect Prescribed Burning Costs. Forest Sci. 43(4), 535–543.

[135-w] González-Cabán, A., Loomis, J.B., Rodriguez, A., Hesseln, H., 2007. A comparison of CVM survey response rates, protests and willingness-to-pay of Native Americans and general population for fuels reduction policies. J. Forest Econ. 13(1), 49–71.

[199-w] González-Cabán, A., McKetta, C.W., 1986. Analyzing Fuel Treatment Costs. West. J. Appl. For. 1(4), 116–121.

[122-w] González-Olabarria, J.R., Palahí, M., Pukkala, T., Trasobares, A., 2008. Optimising the management of Pinus nigra Arn. Stands under endogenous risk of fire in Catalonia. Forest Syst. 17(1), 10-17.

[82-w] González-Olabarria, J.R., Pukkala, T., 2011. Integrating fire risk considerations in landscape-level forest planning. Forest Ecol. Manag. 261(2), 278–287.

[161-w] Gregory, R.S., 2000. Valuing Environmental Policy Options: A Case Study Comparison of Multiattribute and Contingent Valuation Survey Methods. Land Econ. 76(2), 151–173.

[109-w] Gunter, J.E., 1976. Comment on Management Decisions in Severely Damaged Stands. J. Forest. 74(5), 298–299.

[100-w] Haener, M.K., Adamowicz, V.L., 2000. Incorporation of risk in regional forest resource accounts. Ecol. Econ. 33(3), 439–455.

[138-w] Han, H.S., Halbrook, J., Pan, F., Salazar, L., 2010. Economic evaluation of a rolloff trucking system removing forest biomass resulting from shaded fuelbreak treatments. Biomass Bioenerg. 34(7), 1006–1016.

[68-w] Harnden, B.M., Maher, P.M., Martin, G.A., 1973. Forest Fire Detection Systems Design. Manage. Sci. 20(4), 617–628.

[92-w] Hartsough, B.R., Abrams, S., Barbour, R.J., Drews, E.S., McIver, J.D., Moghaddas, J.J., Schwilk, D.W., Stephens, S.L., 2008. The economics of alternative fuel reduction treatments in western United States dry forests: Financial and policy implications from the National Fire and Fire Surrogate Study. For. Policy Econ. 10(6), 344–354.

[49-w] Harry, J., Smith, G., 1971. How much forest protection is needed? Forest. Chron. 47(1), 22–24.

[42-w] Hesseln, H., 2000. The Economics of Prescribed Burning: A Research Review. Forest Sci. 46(3), 322–334.

[180-w] Hesseln, H., 2001. Refinancing and Restructuring Federal Fire Management. J. Forest. 99(11), 4–8.

[11-w] Hesseln, H., Loomis, J.B., González-Cabán, A., 2004. The Effects of Fire on Recreation Demand in Montana. West. J. Appl. For. 19(1), 47–53.

[15-w] Hesseln, H., Loomis, J.B., González-Cabán, A., 2004. Comparing the economic effects of fire on hiking demand in Montana and Colorado. J. Forest Econ. 10(1), 21–35.

[12-w] Hesseln, H., Loomis, J.B., González-Cabán, A., Alexander, S., 2003. Wildfire effects on hiking and biking demand in New Mexico: a travel cost study. J. Environ. Manage. 69(4), 359–368.

[96-w] Hilger, J., Englin, J., 2009. Utility theoretic semi-logarithmic incomplete demand systems in a natural experiment: Forest fire impacts on recreational values and use. Resour. Energ. Econ. 31(4), 287–298.

[61-w] Hjerpe, E E., Kim, Y.S., 2008. Economic Impacts of Southwestern National Forest Fuels Reductions. J. Forest. 106(6), 311–316.

[94-w] Huang, C.H., Finkral, A., Sorensen, C., Kolb, T., 2013. Toward full economic valuation of forest fuels-reduction treatments. J. Environ. Manage. 130, 221–231.

[125-w] Huang, C.H., Sorensen, C., 2011. The Economic Value of Selling Carbon Credits from Restored Forests: A Case Study from the Navajo Nation’s Tribal Forests. West. J. Appl. For. 26(1), 37–45.

[194-w] Hubert, C.A., 1959. The Economics of adequate forest fire protection. Woodland Section. Pulp Paper-Canada. 154-159.

[163-w] Huggett, Jr.R.J., Abt, K.L., Shepperd, W., 2008. Efficacy of mechanical fuel treatments for reducing wildfire hazard. For. Policy Econ. 10(6), 408–414.

[63-w] Hummel, S., Calkin, D.E., 2005. Costs of landscape silviculture for fire and habitat management. Forest Ecol. Manag. 207(3), 385–404.

[172-w] Hystad, P.W., Keller, P.C., 2008. Towards a destination tourism disaster management framework: Long-term lessons from a forest fire disaster. Tourism Manage. 29(1),151–162.

[173-w] Hystad, P., Keller, P., 2006. Disaster Management: Kelowna Tourism Industry’s Preparedness, Impact and Response to a 2003 Major Forest Fire. J. Hosp. Tourism Manage. 13(1), 44–58.

[210-w] Hyytiäinen, K., Haight, R.G., 2010. Evaluation of forest management systems under risk of wildfire. Eur.J. Forest. Res. 129. 909-919.

[144-w] Ince, P.J., Spelter, H., Skog, K.E., Kramp, A., Dykstra, D.P., 2008. Market impacts of hypothetical fuel treatment thinning programs on federal lands in the western United States. For. Policy Econ. 10(6), 363–372.

[56-w] Insley, M., Lei, M., 2007. Hedges and Trees: Incorporating Fire Risk into Optimal Decisions in Forestry Using a No-Arbitrage Approach. J. Agr. Resour. Econ. 32(3), 492–514.

[69-w] Jewell, W.S., 1963. Forest Fire Problems-A Progress Report. Operations Research. 11(5), 678–692.

[146-w] Jones, G., Loeffler, D., Butler, E., Hummel, S., Chung, W., 2013. The financial feasibility of delivering forest treatment residues to bioenergy facilities over a range of diesel fuel and delivered biomass prices. Biomass Bioenerg. 48, 171–180.

[87-w] Kaval, P., 2009. Perceived and actual wildfire danger: An economic and spatial analysis study in Colorado (USA). J. Environ. Manage. 90(5), 1862–1867.

[99-w] Kaval, P., Loomis, J., Seidl, A., 2007. Willingness-to-pay for prescribed fire in the Colorado (USA) wildland urban interface. For. Policy Econ. 9(8), 928–937.

[128-w] Keegan, III C.E., Fiedler, C., Morgan, T.A., 2004. Wildfire in Montana: Potential hazard reduction and economic effects of a strategic treatment program. Forest Prod. J. 54(7-8), 21–25.

[181-w] Kim, Y.S., Wells, A., 2005. The Impact of Forest Density on Property Values. J. Forest. 103(3), 146–151.

[168-w] Kocoloski, M., Griffin, W.M., Matthews, H.S., 2011. Estimating national costs, benefits, and potential for cellulosic ethanol production from forest thinnings. Biomass Bioenerg. 35(5), 2133–2142.

[62-w] Konoshima, M., Albers, H.J., Montgomery, C.A., Arthur, J.L., 2010. Optimal spatial patterns of fuel management and timber harvest with fire risk. Can. J. Forest Res. 40(1), 95–108.

[58-w] Konoshima, M., Montgomery, C.A., Albers, H.J., Arthur, J.L., 2008. Spatial-Endogenous Fire Risk and Efficient Fuel Management and Timber Harvest. Land Econ. 84(3), 449–468.

[182-w] Kountouris, Y., Remoundou, K., 2011. Valuing the Welfare Cost of Forest Fires: a Life Satisfaction Approach. Kyklos. 64(4), 556–578.

[110-w] Lee, Y., Fried, J.S., Albers, H.J., Haight, R.G., 2013. Deploying initial attack resources for wildfire suppression: spatial coordination, budget constraints, and capacity constraints. Can. J. Forest Res. 43(1), 56–65.

[115-w] Liang, J., Calkin, D.E., Gebert, K.M., Venn, T.J., Silverstein, R.P., 2008. Factors influencing large wildland fire suppression expenditures. Int. J. Wildland Fire. 17(5), 650–659.

[75-w] Liu, Z., He, H.S., Chang, Y., Hu, Y., 2010. Analyzing the effectiveness of alternative fuel reductions of a forested landscape in Northeastern China. Forest Ecol. Manag. 259(7), 1255–1261.

[132-w] Loomis, J., 2004. Do nearby forest fires cause a reduction in residential property values? J. Forest Econ. 10(3), 149–157.

[159-w] Loomis, J.B., Bair, L.S., González-Cabán, A., 2002. Language-Related Differences in a Contingent Valuation Study: English versus Spanish. Am. J. Agr. Econ. 84(4), 1091–1102.

[171-w] Loomis, J., Ellingson, L., González-Cabán, A., Seidi, A., 2006. The Role of Ethnicity and Language in Contingent Valuation Analysis: A Fire Prevention Policy Application. Am. J. Econ. Sociol. 65(3), 559–586.

[134-w] Loomis, J.B., González-Cabán, A., 1997. Comparing the Economic Value of Reducing Fire Risk to Spotted Owl Habitat in California and Oregon. Forest Sci. 43(4), 473–482.

[130-w] Loomis, J.B., González-Cabán, A., 1998. A willingness-to-pay function for protecting acres of spotted owl habitat from fire. Ecol. Econ. 25(3), 315–322.

[118-w] Loomis, J., González-Cabán, A., 2010. Forest Service Use of Nonmarket Valuation in Fire Economics: Past, Present, and Future. J. Forest. 108(8), 389–396.

[10-w] Loomis, J., González-Cabán, A., Englin, J., 2001. Testing for Differential Effects of Forest Fires on Hiking and Mountain Biking Demand and Benefits. J. Agr. Resour. Econ. 26(2), 508–522.

[139-w] Loomis, J., Griffin, D., Wu, E., González-Cabán, A., 2002. Estimating the economic value of big game habitat production from prescribed fire using a time series approach. J. Forest Econ. 8(2), 119–129.

[198-w] Loomis, J., González-Cabán, A., 1994. Estimating the Value of Reducing Fire Hazards to Old Growth Forests in the Pacific Northwest: A Contingent Valuation Approach. Int. J. Wildland Fire. 4(4), 209-216.

[98-w] Loomis, J.B., Hung, L.T., González-Cabán, A., 2009. Willingness to pay function for two fuel treatments to reduce wildfire acreage burned: A scope test and comparison of White and Hispanic households. For. Policy Econ. 11(3), 155–160.

[91-w] Loomis, J.B., Le H.T., González-Cabán, A., 2005. Testing transferability of willingness to pay for forest fire prevention among three states of California, Florida and Montana. J. Forest Econ. 11(3), 125–140.

[157-w] Loureiro, M.L., Dominguez Arcos, F., 2012. Applying Best–Worst Scaling in a stated preference analysis of forest management programs. J. Forest Econ. 18(4), 381–394.

[20-w] Loveridge, E.W., 1944. The Fire Suppression Policy of the U. S. Forest Service. J. Forest. 42(8), 549–554.

[107-w] Lowell, E.C., Becker, D.R., Rummer, R., Larson, D., Wadleigh, L., 2008. An Integrated Approach to Evaluating the Economic Costs of Wildfire Hazard Reduction through Wood Utilization Opportunities in the Southwestern United States. Forest Sci. 54(3), 273–283.

[45-w] Lynch, D.L., 2004. What Do Forest Fires Really Cost? J. Forest. 102(6), 42–49.

[188-w] Mangan, R.J., 2001. Issues in reducing costs on large wildfires. Fire. Manage. Today. 61(3), 6-10.

[43-w] Martell, D.L., 1980. The optimal rotation of a flammable forest stand. Can. J. Forest Res. 10(1), 30–34.

[50-w] Martell, D.L., 1994. The impact of fire on timber supply in Ontario. Forest. Chron. 70(2), 164–173.

[64-w] Martell, D.L., Drysdale, R.J., Doan, G.E., Boychuk, D., 1984. An Evaluation of Forest Fire Initial Attack Resources. Interfaces. 14(5), 20–32.

[189-w] Martin, R.E., Cooper, R.W., Bigler Crow, A., Cuming, J.A., Phillips, C.B., 1977. Report of Task Force on Prescribed Burning. J. Forest. 75(5), 297-301.

[151-w] Mason, C.L., Lippke, B.R., Zobrist, K.W., Bloxton, T.D., Ceder, K.R., Comnick, J.M., McCarter, J.B., Rogers, H.K., 2006. Investments in Fuel Removals to Avoid Forest Fires Result in Substantial Benefits. J. Forest. 104(1), 27–31.

[93-w] Mavsar, R., González-Cabán, A., Varela, E., 2013. The state of development of fire management decision support systems in America and Europe. For. Policy Econ. 29, 45–55.

[95-w] Mavsar, R., Japelj, A., Kovac, M., 2013. Trade-offs between fire prevention and provision of ecosystem services in Slovenia. For. Policy Econ. 29, 62–69.

[190-w] McKee, M.C., Berrens, R.P., Jones, M., Helton, R., Talberth, J., 2004. Using Experimental Economics to Examine Wildfire Insurance and Averting Decisions in the Wildland–Urban Interface. Soc. Natur. Resour. 17(6), 491-507.

[206-w] McIver, J.D., Adams, P.W., Doyal, J.A., Drews, E.S., Hartsough, B.R., Kellogg, L.D., Niwa, C.G., Ottmar, R., Peck, R., Taratoot, M., Torgersen, T., Youngblood, A., 2003. Environmental Effects and Economics of Mechanized Logging for Fuel Reduction in Northeastern Oregon Mixed-Conifer Stands. West. J. Appl. For. 18(4), 238–249.

[111-w] McKetta, C.W., González-Cabán, A., 1985. Economic Costs of Fire-Suppression Forces. J. Forest. 83(7), 429–432.

[73-w] Mendes, I., 2010. A theoretical economic model for choosing efficient wildfire suppression strategies. For. Policy Econ. 12(5), 323–329.

[4-w] Mercer, D.E., Prestemon, J.P., 2005. Comparing production function models for wildfire risk analysis in the wildland–urban interface. For. Policy Econ. 7(5), 782–795.

[1-w] Mercer, D.E., Prestemon, J.P., Butry, D.T., Pye, J.M., 2007. Evaluating Alternative Prescribed Burning Policies to Reduce Net Economic Damages from Wildfire. Am. J. Agr. Econ. 89(1), 63–77.

[36-w] Mills, T.J., Flowers, P.J., 1985. Fire-induced changes in net value of timber: a sensitivity analysis. Can. J. Forest Res. 15(5), 973–981.

[46-w] Mills, T.J., Flowers, P.J., 1986. Wildfire Impacts on the Present Net Value of Timber Stands: Illustrations in the Northern Rocky Mountains. Forest Sci. 32(3), 707–724.

[35-w] Moak, J.E., 1976. Fire Prevention: Does it Pay? J. Forest. 74(9), 612–614.

[154-w] Moeltner, K., Kim, M.K., Zhu, E., Yang, W., 2013. Wildfire smoke and health impacts: A closer look at fire attributes and their marginal effects. J. Environ. Econ. Manag. 66(3), 476–496.

[207-w] Montgomery, C.A., Brodie, J.D., Cleaves, D.A., 1986. Allowable cut effect and fire-damage appraisal. West. J. Appl. For. 1, 100-103.

[153-w] Morgan, T.A., Brandt, J.P., Baldridge, J.D., Loeffler, D.R., 2011. Use of Financial and Economic Analyses by Federal Forest Managers for Woody Biomass Removal. West. J. Appl. For. 26(1), 5–12.

[39-w] Morrell, F., 1931. The 25-25-50 Ratio in Financing Fire Protection. J. Forest. 29(4), 520–523.

[191-w] Moseley, C., Toth, N., 2004. Fire Hazard Reduction and Economic Opportunity: How Are the Benefits of the National Fire Plan Distributed? Soc. Natur. Resour. 17(8), 701–716.

[201-w] Murnane, R.R., 2006. Catastrophe Risk models for wildfires in the Wildland-urban Interface: What insurers need. Nat. Hazards Rev. 7(4), 150-156.

[31-w] Nautiyal, J.C., Doan, G.E., 1974. Economics of Forest Fire Control: Trading Planned Cut for Protection Expenditure. Can. J. Forest Res. 4(1), 82–90.

[116-w] Nelson, T.C., 1979. Fire Management Policy in the National Forests–A New Era. J. Forest. 77(11), 723–725.

[152-w] Nielsen-Pincus, M., Moseley, C., Gebert, K., 2013. The Effects of Large Wildfires on Employment and Wage Growth and Volatility in the Western United States. J. Forest. 111(6), 404–411.

[142-w] Nielsen-Pincus, M., Moseley, C., Gebert, K., 2014. Job growth and loss across sectors and time in the western US: The impact of large wildfires. For. Policy Econ. 38, 199–206.

[208-w] North, D.W., Offensend, F.L., Smart, C.N., 1975. Planning wildfire protection for the Santa Monica mountains: an economic analysis of alternatives. Fire J. 69-78.

[22-w] Noste, N.V., Davis, J.B., 1975. A Critical Look at Fire Damage Appraisal. J. Forest. 73(11), 715–719.

[102-w] Ntaimo, L., Gallego Arrubla, J.A., Stripling, C., Young, J., Spencer, T., 2012. A stochastic programming standard response model for wildfire initial attack planning. Can. J. Forest Res. 42(6), 987–1001.

[162-w] Ohlson, D.W., Berry, T.M., Gray, R.W., Blackwell, B.A., Hawkes, B.C., 2006. Multi-attribute evaluation of landscape-level fuel management to reduce wildfire risk. For. Policy Econ. 8(8), 824–837.

[65-w] Parks, G.M., 1964. Development and Application of a Model for Suppression of Forest Fires. Manage. Sci. 10(4), 760–766.

[200-w] Pasalodos-tato, M., Pukkala, T., Roja Alboreca, A., 2010. Optimal management of Pinus pinaster in galicia (Spain) under risk of fire. Int. J. Wildland Fire. 19, 937-948.

[166-w] Polagye, B.L., Hodgson, K.T., Malte, P.C., 2007. An economic analysis of bio-energy options using thinnings from overstocked forests. Biomass. Bioenerg. 31(2–3), 105–125.

[81-w] Prante, T., Little, J.M., Jones, M.L., McKee, M., Berrens, R.P., 2011. Inducing private wildfire risk mitigation: Experimental investigation of measures on adjacent public lands. J. Forest Econ. 17(4), 415–431.

[129-w] Prebble, M.L., Gardiner, L.M., 1958. Degrade and value loss in fire-killed pine in the Mississagi area of Ontario. Forest. Chron. 34(2), 139–158.

[89-w] Prestemon, J.P., Abt, K.L., Barbour, R.J., 2012. Quantifying the net economic benefits of mechanical wildfire hazard treatments on timberlands of the western United States. For. Policy Econ. 21, 44–53.

[143-w] Prestemon, J.P., Abt, K.L., Huggett, Jr.R.J., 2008. Market impacts of a multiyear mechanical fuel treatment program in the U.S. For. Policy Econ. 10(6), 386–399.

[67-w] Prestemon, J.P., Donovan, G.H., 2008. Forecasting Resource-Allocation Decisions under Climate Uncertainty: Fire Suppression with Assessment of Net Benefits of Research. Am. J. Agr. Econ. 90(4), 1118–1129.

[6-w] Prestemon, J.P., Wear, D.N., Stewart, F.J., Holmes, T.P., 2006. Wildfire, timber salvage, and the economics of expediency. For. Policy Econ. 8(3), 312–322.

[9-w] Reed, W.J., 1984. The effects of the risk of fire on the optimal rotation of a forest. J. Environ. Econ. Manag. 11(2), 180–190.

[183-w] Reed, W.J., 1987. Protecting a forest against fire: optimal protection patterns and harvest policies. Nat. Resour. Model. 2(1), 23-53.

[33-w] Reed, W.J., Apaloo, J., 1991. Evaluating the effects of risk on the economics of juvenile spacing and commercial thinning. Can. J. Forest Res. 21(9), 1390–1400.

[52-w] Reed, W.J., Errico, D., 1985. Assessing the long-run yield of a forest stand subject to the risk of fire. Can. J. Forest Res. 15(4), 680–687.

[204-w] Rideout, D., Loomis, J., Ziesler, P., Wei, Y., 2012. Comparing fire protection and improvement values at four major us national parks and assessing the potential for generalized value categories. Int. J. Safety Security Eng. 2(1), 1-12.

[26-w] Rideout, D.B., Omi, P.N., 1990. Alternate Expressions for the Economic Theory of Forest Fire Management. Forest Sci. 36(3), 614–624.

[32-w] Rideout, D.B., Omi, P.N., 1995. Estimating the Cost of Fuels Treatment. Forest Sci. 41(4), 664–674.

[97-w] Rideout, D.B., Ziesler, P.S., Kernohan, N.J., 2014. Valuing fire planning alternatives in forest restoration: Using derived demand to integrate economics with ecological restoration. J. Environ. Manage. 141, 190–200.

[158-w] Rideout, D.B., Ziesler, P.S., Kling, R., Loomis, J.B., Botti, S.J., 2008. Estimating rates of substitution for protecting values at risk for initial attack planning and budgeting. For. Policy Econ. 10(4), 205–219.

[140-w] Riera, P., Mogas, J., 2004. Evaluation of a risk reduction in forest fires in a Mediterranean region. For. Policy Econ. 6(6), 521–528.

[133-w] Rittmaster, R., Adamowicz, W.L., Amiro, B., Pelletier, R.T., 2006. Economic analysis of health effects from forest fires. Can. J. Forest Res. 36(4), 868–877.

[186-w] Rodriguez y Silva F., González-Cabán, A., 2010. ‘SINAMI’: a tool for the economic evaluation of forest fire management programs in Mediterranean ecosystems. Int. J. Wildland Fire. 19, 927-936.

[78-w] Rodríguez y Silva, F., Molina, J.R., González-Cabán, A., Herrera Machuca, M.Á., 2012. Economic vulnerability of timber resources to forest fires. J. Environ. Manage. 100, 16–21.

[83-w] Román, M.V., Azqueta, D., Rodrígues, M., 2013. Methodological approach to assess the socio-economic vulnerability to wildfires in Spain. Forest Ecol. Manag. 294, 158–165.

[76-w] Rummer, B., 2008. Assessing the cost of fuel reduction treatments: A critical review. For. Policy Econ. 10(6), 355–362.

[174-w] Rytwinski, A., Crowe, K.A., 2010. A simulation-optimization model for selecting the location of fuel-breaks to minimize expected losses from forest fires. Forest Ecol. Manag. 260(1), 1–11.

[29-w] Sackett, S.S., Webster, H.H., Lord, W.B., 1967. Economic Guides for Allocating Forest Fire Protection Budgets in Wisconsin. J. Forest. 65(9), 636–641.

[119-w] Sessions, J., Bettinger, P., Buckman, R., Newton, M., Hamann, J., 2004. Hastening the Return of Complex Forests Following Fire: The Consequences of Delay. J. Forest. 102(3), 38–45.

[184-w] Shepherd, C., Grimsrud, C., Berrens, R.P., 2009. Determinants of National Fire Plan Fuels Treatment Expenditures: A Revealed Preference Analysis for Northern New Mexico. Environ. Manage. 44, 776-788.

[209-w] Sparhawk, W.N., 1925. The use of liability ratings in planning forest fire protection. J. Agric. Res. 30(8), 693-762.

[126-w] Snider, G., Daugherty, P.J., Wood, D., 2006. The Irrationality of Continued Fire Suppression: An Avoided Cost Analysis of Fire Hazard Reduction Treatments Versus No Treatment. J. Forest. 104(8), 431–437.

[169-w] Soliño, M., 2010. External benefits of biomass-e in Spain: An economic valuation. Bioresource Technol. 101(6), 1992–1997.

[136-w] Soliño, M., Prada, A., Vázquez, M.X., 2010. Designing a forest-energy policy to reduce forest fires in Galicia (Spain): A contingent valuation application. J. Forest Econ. 16(3), 217–233.

[86-w] Spring, D.A., Kennedy, J.O.S., 2005. Existence value and optimal timber wildlife management in a flammable multistand forest. Ecol. Econ. 55(3), 365–379.

[85-w] Spring, D.A., Kennedy, J., Lindenmayer, D.B., McCarthy, M.A., McNally, R., 2008. Optimal management of a flammable multi-stand forest for timber production and maintenance of nesting sites for wildlife. Forest Ecol. Manag. 255(11), 3857–3865.

[90-w] Starbuck, C.M., Berrens, R.P., McKee, M., 2006. Simulating changes in forest recreation demand and associated economic impacts due to fire and fuels management activities. For. Policy Econ. 8(1), 52–66.

[192-w] Steele, T.W., Stier, J.C., 1998. An economic evaluation of public and organized wildfire detection in Wisconsin. Int. J. Wildland Fire. 8(4), 205-215.

[145-w] Stetler, K.M., Venn, T.J., Calkin, D.E., 2010. The effects of wildfire and environmental amenities on property values in northwest Montana, USA. Ecol. Econ. 69(11), 2233–2243.

[80-w] Stockmann, K., Burchfield, J., Calkin, D., Venn, T., 2010. Guiding preventative wildland fire mitigation policy and decisions with an economic modeling system. For. Policy Econ. 12(2), 147–154.

[197-w] Sullivan, J., Omi, P.N., Dyer, A.A., González-Cabán, A., 1987. Evaluating the Economic Efficiency of Wildfire Rehabilitation Treatments. West. J. Appl. For. 2(2), 58–61.

[155-w] Sundstrom, S., Nielsen-Pincus, M., Moseley, C., McCaffery, S., 2012. Woody Biomass Use Trends, Barriers, and Strategies: Perspectives of US Forest Service Managers. J. Forest. 110(1), 16–24.

[27-w] Sutherland, C.F., 1973. Cost of Forest Closure in Two Oregon Counties. J. Forest. 71(10), 644–647.

[185-w] Stollery, K.R., 2005. Climate change and optimal rotation in a flammable forest. Nat. Resour. Model. 18(1), 91-112.

[23-w] Teeter, L.D., Dyer, A.A., 1986. A Multiattribute Utility Model for Incorporating Risk in Fire Management Planning. Forest Sci. 32(4), 1032–1048.

[193-w] Temple, S., 2011. Forestation and its discontents: the invention of an uncertain landscape in Southwestern France, 1850-Present. **Environ Hist**. 17, 13-34.

[165-w] Varela, E., Bredahl Jacobsen, J., Soliño, M., 2014. Understanding the heterogeneity of social preferences for fire prevention management. Ecol. Econ. 106, 91–104.

[48-w] Vasievich, J.M., 1980. Costs of Hazard-Reduction Burning on Southern National Forests. South. J. Appl. For. 4(1), 12–15.

[202-w] Vegh, T., Huang, C.H., Finkral, A., 2013. Carbon Credit Possibilities and Economic Implications of Fuel Reduction Treatments. West. J. Appl. For. 28(2), 57–65.

[103-w] Venn, T.J., Calkin, D.E., 2011. Accommodating non-market values in evaluation of wildfire management in the United States: challenges and opportunities. Int. J. Wildland Fire. 20(3), 327–339.

[38-w] Wade, D.D., Ward, D.E., 1975. Management Decisions in Severely Damaged Stands. J. Forest. 73(9), 573–577.

[17-w] Wagner, C.E. Van., 1979. The Economic Impact of Individual Fires on the Whole Forest. Forest. Chron. 55(2), 47–50.

[77-w] Walker, S.H., Rideout, D.B., Loomis, J.B., Reich, R., 2007. Comparing the value of fuel treatment options in northern Colorado’s urban and wildland–urban interface areas. For. Policy Econ. 9(6), 694–703.

[108-w] Wei, Y., Rideout, D., Kirsch, A., 2008. An optimization model for locating fuel treatments across a landscape to reduce expected fire losses. Can. J. Forest Res. 38(4), 868–877.

[149-w] Winter, G.J., Fried, J.S., 2001. Estimating Contingent Values for Protection from Wildland Fire Using a Two-Stage Decision Framework. Forest Sci. 47(3), 349–360.

[30-w] Worrell, A.C., 1955. Economics of Fire Detection in the South. J. Forest. 53(9), 639–644.

[88-w] Wu, T., Kim, Y.S., 2013. Pricing ecosystem resilience in frequent-fire ponderosa pine forests. For. Policy Econ. 27, 8–12.

[54-w] Yoder, J., 2003. The economic logic of prescribed burning law and regulation. J. Range Manage. 56(4), 306–313.

[57-w] Yoder, J., 2004. Playing with Fire: Endogenous Risk in Resource Management. Am. J. Agr. Econ. 86(4), 933–948.

[37-w] Yoder, J., Blatner, K., 2004. Incentives and Timing of Prescribed Fire for Wildfire Risk Management. J. Forest. 102(6), 38–41.

[14-w] Yoder, J., Tilley, M., Engle, D., Fuhlendorf, S., 2003. Economics and Prescribed Fire Law in the United States. Rev. Agr. Econ. 25(1), 218–233.

[156-w] Zamora, R., Molina-Martínez, J.R., Herrera, M.A., Rodríguez y Silva, F., 2010. A model for wildfire prevention planning in game resources. Ecol. Model. 221(1), 19–26.

**Pests** n_2_=65.

[29-pe] Asaro, C., Carter, D.R., Berisford, C.W., 2006. Control of Low-Level Nantucket Pine Tip Moth Populations: A Cost-Benefit Analysis. South. J. Appl. For. 30(4), 182–187.

[12-pe] Anderson, W.C., Guldin, R.W., Vasievich, J.M., 1987. Assessing the Risk of Insect Attack in Plantation Investments. J. Forest. 85(1), 46–47.

[54-pe] Bergseng, E., Økland, B., Gobakken, T., Magnusson, C., Rafoss, T., Solberg, B., 2012. Combining ecological and economic modelling in analysing a pest invasion contingency plan - The case of pine wood nematode in Norway. Scand. J. Forest Res. 27(4), 337–349.

[45-pe] Bigsby, K.M., Ambrose, M.J., Tobin, P.C., Sills, E.O., 2014. The cost of gypsy moth sex in the city. Urban For. Urban Gree. 3(13), 459-468.

[40-pe] Bogle, T., Kooten, G.C. Van., 2012. Why mountain pine beetle exacerbates a principal-agent relationship: exploring strategic policy responses to beetle attack in a mixed species forest. Can. J. Forest Res. 42(3), 621–630.

[52-pe] Bogle, T., Kooten, G.C. Van., 2013. Options for maintaining forest productivity after natural disturbance: A principal–agent approach. For. Policy Econ. 26, 138–144.

[19-pe] Chang, W.Y., Lantz, V.A., Hennigar, C.R., MacLean, D.A., 2012. Benefit-cost analysis of spruce budworm (Choristoneura fumiferana Clem.) control: Incorporating market and non-market values. J. Environ. Manage. 93(1), 104–112.

[30-pe] Chang, W.Y., Lantz, V.A., Hennigar, C.R., MacLean, D.A., 2012. Economic impacts of forest pests: a case study of spruce budworm outbreaks and control in New Brunswick, Canada. Can. J. Forest Res. 42(3), 490–505.

[55-pe] Chang, W.Y., Lantz, van A., Mc Lean, D.A., 2011. Social Benefits of Controlling Forest Insect Outbreaks: A Contingent Valuation Analysis in Two Canadian Provinces. Can. J. Agr. Econ. 59, 383-404.

[28-pe] Clarke, S.R., Billings, R.F., 2003. Analysis of the Southern Pine Beetle Suppression Program on the National Forests in Texas in the 1990s. South. J. Appl. For. 27(2), 122–129.

[41-pe] Conway, B.E., Leefers, L.A., McCullough, D.G., 1999. Yield and financial losses associated with a jack pine budworm outbreak in Michigan and the implications for management. Can. J. Forest Res. 29(3), 382–392.

[44-pe] Coulston, J.W., Koch, F.H., Smith, W.D., Sapio, F.J., 2008. Invasive forest pest surveillance: survey development and reliability. Can. J. Forest Res. 38(9), 2422–2433.

[34-pe] Eaton, C.B., 1962. Entomological Considerations in the Economics of Forest Pest Control. J. Forest. 60(5), 309–311.

[21-pe] Gatto, P., Zocca, A., Battisti, A., Barrento, M.J., Branco, M., Paiva, M.R., 2009. Economic assessment of managing processionary moth in pine forests: A case-study in Portugal. J. Environ. Manage. 90(2), 683–691.

[5-pe] Herrick, O.W., 1981. Forest Pest Management Economics–Application to the Gypsy Moth. Forest Sci. 27(1), 128–138.

[1-pe] Holmes, T.P., 1991. Price and welfare effects of catastrophic forest damage from Southern pine beetle epidemics. Forest Sci. 37(2), 500–516.

[16-pe] Holmes, T.P., Liebhold, A.M., Kovacs, K.F., Von Holle, B., 2010. A spatial-dynamic value transfer model of economic losses from a biological invasion. Ecol. Econ. 70(1), 86–95.

[42-pe] Holmes, T.P., Murphy, E.A., Bell, K.P., 2006. Exotic forest insects and residential property values. Agr. Resource Econ. Rev. 35(1), 155-166.

[43-pe] Holmes, T.P., Murphy, E.A., Bell, K.P., Royle, D.D., 2010. Property Value Impacts of Hemlock Woolly Adelgid in Residential Forests. Forest Sci. 56(6), 529–540.

[25-pe] Huitu, O., Kiljunen, N., Korpimäki, E., Koskela, E., Mappes, T., Pietiäinen, H., Pöysä, H., Henttonen, H., 2009. Density-dependent vole damage in silviculture and associated economic losses at a nationwide scale. Forest Ecol. Manag. 258(7), 1219–1224.

[6-pe] Irland, L.C., 1980. Pulpwood, pesticides, and people. Controlling spruce budworm in northeastern North America. Environ. Manage. 4(5), 381–389.

[38-pe] Johnson, N.E., 1963. Some economic considerations in planning control of insects affecting young forest trees. J. Forest. 61(6), 426–429.

[20-pe] Kovacs, K.F., Haight, R.G., McCullough, D.G., Mercader, R.J., Siegert, N.W., Liebhold, A.M., 2010. Cost of potential emerald ash borer damage in U.S. communities, 2009–2019. Ecol. Econ. 69(3), 569–578.

[15-pe] Kovacs, K.F., Haight, R.G., Mercader, R.J., McCullough, D.G., 2014. A bioeconomic analysis of an emerald ash borer invasion of an urban forest with multiple jurisdictions. Resour. Energ. Econ. 36(1), 270–289.

[24-pe] Kovacs, K.F., Mercader, R.J., Haight, R.G., Siegert, N.W., McCullough, D.G., Liebhold, A.M., 2011. The influence of satellite populations of emerald ash borer on projected economic costs in U.S. communities, 2010–2020. J. Environ. Manage. 92(9), 2170–2181.

[47-pe] Kumar, A., 2009. A conceptual comparison of bioenergy options for using mountain pine beetle infested wood in Western Canada. Bioresource Technol. 100(1), 387–399.

[49-pe] Kumar, A., Flynn, P., Sokhansanj, S., 2008. Biopower generation from mountain pine infested wood in Canada: An economical opportunity for greenhouse gas mitigation. Renew. Energ. 33(6), 1354–1363.

[65-pe] Leuschner, W.A., Maine, J.D., 1980. Estimating the Southern Pine Beetle’s Grazing Impact. Bull Entomol Soc Am. 26(2), 117–120.

[64-pe] Leuschner, W.A., Max, T.A., Spitte, G.D., Wisdom, H.W., 1978. Estimating southern pine beetle timber damages. Bull Entomol Soc Am. 24(1), 29-34.

[58-pe] Leuschner, W.A., Newton, C.M., 1974. Benefits of Forest Insect Control. Bull Entomol Soc Am. 20(3), 223-227.

[8-pe] Leuschner, W.A., Young, R.L., 1978. Estimating the Southern Pine Beetle’s Impact on Reservoir Campsites. Forest Sci. 24(4), 527–537.

[37-pe] Leuschner, W.A., Young, J.A., Waldon, S.A., Ravlin, F.W., 1996. Potential Benefits of Slowing the Gypsy Moth’s Spread. South. J. Appl. For. 20(2), 65–73.

[61-pe] Li, R., Buongiorno, J., Zhu, S., Turner, J.A., Prestemon, J., 2007. Potential economic impact of limiting the international trade of timber as a phytosanitary measure. Int. For. Rev. 9(1), 514-525.

[4-pe] Liebhold, A.M., Berck, P., Williams, N.A., Wood, D.L., 1986. Estimating and Valuing Western Pine Beetle Impacts. Forest Sci. 32(2), 325–338.

[17-pe] Marten, A.L., Moore, C.C., 2011. An options based bioeconomic model for biological and chemical control of invasive species. Ecol. Econ. 70(11), 2050–2061.

[7-pe] Marty, R.J., Alison, G.R., 1960. Appraising White-Pine Weevil Control Opportunities. J. Forest. 58(3), 203–206.

[59-pe] Mathey, A.H., Nelson, H., 2010. Assessing forest management strategies under a mountain pine beetle attack in Alberta: exploring the impacts. Can. J. Forest Res. 40(4), 597–610.

[60-pe] Mc Donalds, H., Mc Kenney, D.W., Nealis, V., 1997. A Bug Is a Bug Is a Bug: Symbolic Responses to Contingent Valuation Questions about Forest Pest Control Programs? Can. J. Agr. Econ. 45, 145-163.

[35-pe] McCullough, D.G., Mercader, R.J., 2012. Evaluation of potential strategies to SLow Ash Mortality (SLAM) caused by emerald ash borer (Agrilus planipennis): SLAM in an urban forest. Int. J. Pest Manage. 58(1), 9–23.

[2-pe] Michalson, E.L., 1975. Economic impact of mountain pine beetle on outdoor recreation. South. J. Agr. Econ. 7(2), 43–50.

[62-pe] Miller, J.D., Lindsay, B.E., 1993. Willingness to pay for a state gypsy moth control program in New Hampshire: a contigent valuation case study. Forest Entomol. 86(3), 828-837.

[14-pe] Moore, C.C., Holmes, T.P., Bell, K.P., 2011. An attribute-based approach to contingent valuation of forest protection programs. J. Forest Econ. 17(1), 35–52.

[56-pe] Moore, C.C., Phaneuf, D.J., Thurman, W.N., 2011. A Bayesian Bioeconometric Model of Invasive Species Control: The Case of the Hemlock Woolly Adelgid. Environ. Resour. Econ. 50(1), 1-26.

[33-pe] Newport, C.A., 1962. Economics of Forest Pest Control. J. Forest. 60(5), 306–308.

[32-pe] Orbay, L., McLean, J.A., Sauder, B.J., Cottell, P.L., 1994. Economic losses resulting from ambrosia beetle infestation of sawlogs in coastal British Columbia, Canada. Can. J. Forest Res. 24(6), 1266–1276.

[11-pe] Patriquin, M.N., Wellstead, A.M., White, W.A., 2007. Beetles, trees, and people: Regional economic impact sensitivity and policy considerations related to the mountain pine beetle infestation in British Columbia, Canada. For. Policy Econ. 9(8), 938–946.

[27-pe] Prestemon, J.P., Abt, K.L., Potter, K.M., Koch, F.H., 2013. An Economic Assessment of Mountain Pine Beetle Timber Salvage in the West. West. J. Appl. For. 28(4), 143–153.

[46-pe] Price, J.I., McCollum, D.W., Berrens, R.P., 2010. Insect infestation and residential property values: A hedonic analysis of the mountain pine beetle epidemic. For. Policy Econ. 12(6), 415–422.

[9-pe] Reed, W.J., Errico, D., 1987. Techniques for assessing the effects of pest hazards on long-run timber supply. Can. J. Forest Res. 17(11), 1455–1465.

[31-pe] Rose, D.W., 1974. Economic Implications of Stocking and Budworm Attacks for Jack Pine Management. Can. J. Forest Res. 4(3), 349–360.

[48-pe] Rossi, F.J., Carter, D.R., Alavalapati, J.R.R., Nowak, J.T., 2011. Assessing landowner preferences for forest management practices to prevent the southern pine beetle: An attribute-based choice experiment approach. For. Policy Econ. 13(4), 234–241.

[13-pe] Rumpf, D.L., Melachrinoudis, E., Rumpf, T., 1985. Improving Efficiency in a Forest Pest Control Spray Program. Interfaces. 15(5), 1–11.

[10-pe] Sims, C., Aadland, D., Finnoff, D., 2010. A dynamic bioeconomic analysis of mountain pine beetle epidemics. J. Econ. Dyn. Control. 34(12), 2407–2419.

[36-pe] Schwab, O., Maness, T., Bull, G., Roberts, D., 2009. Modeling the effect of changing market conditions on mountain pine beetle salvage harvesting and structural changes in the British Columbia forest products industry. Can. J. Forest Res. 39(10), 1806–1820.

[22-pe] Schwab, O., Maness, T., Bull, G., Welham, C., Seely, B., Blanco, J., 2011. Modeling the timber supply impact of introducing weevil-resistant spruce in British Columbia with cellular automata. For. Policy Econ. 13(1), 61–68.

[57-pe] Sharov, A.A., 2004. Bioeconomics of Managing the Spread of Exotic Pest Species with Barrier Zones, Risk Anal. 24(4), 879-892.

[23-pe] Slaney, G.L., Lantz, V.A., MacLean, D.A., 2009. The economics of carbon sequestration through pest management: application to forested landbases in New Brunswick and Saskatchewan, Canada. For. Policy Econ. 11(7), 525–534.

[18-pe] Slaney, G.L., Lantz, V.A., MacLean, D.A., 2010. Assessing costs and benefits of pest management on forested landbases in eastern and western Canada. J. Forest Econ. 16(1), 19–34.

[53-pe] Strutt, A., Turner, J.A., Haack, R.A., Olson, L., 2013. Evaluating the impacts of an international phytosanitary standard for wood packaging material: Global and United States trade implications. For. Policy Econ. 27, 54–64.

[39-pe] Sydnor, T.D., Bumgardner, M., Todd, A., 2007. The potential economic impacts of emerald ash borer (Agrilus planipennis) on Ohio, U.S., communities. Arboric. Urban. For. 33(1), 48–54.

[3-pe] Thompson, W.A., Holling, C.S., Kira, D., Huang, C.C., Vertinskf, I., 1979. Evaluation of alternative forest system management policies the case of the spruce budworm in New Brunswick. J. Environ. Econ. Manag. 6(1), 51–68.

[63-pe] Vannatta, A.R., Hauer, R.H., Schuettpelz, N.M., 2012. Economic Analysis of Emerald Ash Borer (Coleoptera: Buprestidae) Management Options. J. Econ. Entomol. 105(1), 196–206.

[26-pe] Yemshanov, D., McKenney, D.W., de Groot, P., Haugen, D., Sidders, D., Joss, B., 2009. A bioeconomic approach to assess the impact of an alien invasive insect on timber supply and harvesting: a case study with Sirex noctilio in eastern Canada. Can. J. Forest Res. 39(1), 154–168.

[51-pe] Walsh, R.G., Bjonback, R.D., Aiken, R.A., Rosenthal, D.H., 1990. Estimating the public benefits of protecting forest quality. J. Environ. Manage. 30(2), 175–189.

[50-pe] Watson, A.C., Sullivan, J., Amacher, G.S., Asaro, C., 2013. Cost sharing for pre-commercial thinning in southern pine plantations: Willingness to participate in Virginia’s pine bark beetle prevention program. For. Policy Econ. 34, 65–72.

**Pathogens** n_3_=16.

[5-pa] Anderson, R.L., McClure, J.P., Cost, N., Uhler, R.J., 1986. Estimating Fusiform Rust Losses in Five Southeast States. South. J. Appl. For. 10(4), 237–240.

[12-pa] Baughman, M.J., 1985. Economics of Dutch Elm Disease Control: A Model and Case Study. J. Forest. 83(9), 554–557.

[13-pa] Brawner, J.T., Carter, D.R., Huber, D.A., White, T.L., 1999. Projected gains in rotation age volume and value from fusiform rust resistant slash and loblolly pines. Can. J. Forest Res. 29(6), 737–742.

[4-pa] Bridgwater, F.E., Smith, W.D., 1997. Economic Impact of Fusiform Rust on the Value of Loblolly Pine Plantations. South. J. Appl. For. 21(4), 187–192.

[1-pa] Cubbage, F.W., Pye, J.M., Holmes, T.P., Wagner, J.E., 2000. An Economic Evaluation of Fusiform Rust Protection Research. South. J. Appl. For. 24(2), 77–85.

[9-pa] Dole, D., Brennan, P., 1996. An economic tool for evaluating disease management in the Jarrah forests of Western Australia. Aust. J. Agr. Resour. Ec. 40(3), 235–248.

[10-pa] Haight, R.G., Homans, F.R., Horie, T., Mehta, S.V., Smith, D.J., Venette, R.C., 2011. Assessing the Cost of an Invasive Forest Pathogen: A Case Study with Oak Wilt. Environ. Manage. 47(3), 506–517.

[7-pa] Horie, T., Haight, R.G., Homans, F.R., Venette, R.C., 2013. Optimal strategies for the surveillance and control of forest pathogens: A case study with oak wilt. Ecol. Econ. 86, 78–85.

[15-pa] Ivkovic, M., Baltunis, B., Gapare, W., Sasse, J., Dutkowski, G., Elms, S., Wu, H., 2010. Breeding against dothistroma needle blight of radiata pine in Australia. Can. J. Forest Res. 40(8), 1653–1660.

[14-pa] Kovacs, K., Holmes, T.P., Englin, J.E., Alexander, J., 2011. The Dynamic Response of Housing Values to a Forest Invasive Disease: Evidence from a Sudden Oak Death Infestation. Environ. Resour. Econ. 49(3), 445–471.

[8-pa] Kovacs, K., Václavík, T., Haight, R.G., Pang, A., Cunniffe, N.J., Gilligan, C.A., Meentemeyer, R.K., 2011. Predicting the economic costs and property value losses attributed to sudden oak death damage in California (2010–2020). J. Environ. Manage. 92(4), 1292–1302.

[6-pa] Meldrum, J.R., Champ, P.A., Bond, C.A., 2013. Heterogeneous nonmarket benefits of managing white pine bluster rust in high-elevation pine forests. J. Forest Econ. 19(1), 61–77.

[11-pa] Ndeffo Mbah, M.L., Gilligan, C.A., 2010. Balancing Detection and Eradication for Control of Epidemics: Sudden Oak Death in Mixed-Species Stands. PLoS ONE. 5(9), e12317.

[3-pa] Powers, H.R., McClure, J.P., Knight, H.A., Dutrow, G.F., 1974. Incidence and Financial Impact of Fusiform Rust in the South. J. Forest. 72(7), 398–401.

[2-pa] Redmond, C.H., Anderson, R.L., 1986. Economic Benefits of Using the Resistance Screening Center to Assess Relative Resistance to Fusiform Rust. South. J. Appl. For. 10(1), 34–37.

[16-pa] Wagner, J.E., Holmes, T.P., 1999. Estimating Economic Gains for Landowners Due to Time-Dependent Changes in Biotechnology. Forest Sci. 45(2), 163–170.

**Storms** n_3_=16.

[10-s] Bilek, E.M., Bown, H.E., 1997. Impact of Windthrow Risk on the Management of Forest Estates in the Canterbury Region in New Zealand. J. Forest Plan. 3(2), 83–90.

[5-s] Brunette, M., Couture, S., 2008. Public compensation for windstorm damage reduces incentives for risk management investments. For. Policy Econ. 10(7–8), 491–499.

[16-s] Brunette, M., Couture, S., Laye, J., 2014. Optimising forest management under storm risk with a Markov decision process model. J. Environ. Econ. Policy. 4(2), 141-163.

[15-s] Couture, S., Reynaud, A., 2008. Multi-stand Forest Management under a Climatic Risk: Do Time and Risk Preferences Matter? Environ. Model. Assess. 13(2), 181-193.

[14-s] Gardiner, B.A., Quine, C.P., 2000. Management of forests to reduce the risk of abiotic damage - a review with particular reference to the effects of strong winds. Forest Ecol. Manag. 135, 261-277.

[4-s] Haight, R.G., Smith, W.D., Straka, T.J., 1995. Hurricanes and the Economics of Loblolly Pine Plantations. Forest Sci. 41(4), 675–688.

[13-s] Loisel, P., 2014. Impact of storm risk on Faustmann rotation. For. Policy Econ. 38, 191–198.

[6-s] Marsinko, A.P.C., Straka, T.J., Baumann, J.L., 1993. Hurricane Hugo: A South Carolina Update. J. Forest. 91(9), 9–17.

[7-s] Meilby, H., Strange, N., Jellesmark Thorsen, B., 2001. Optimal spatial harvest planning under risk of windthrow. Forest Ecol. Manag. 149(1–3), 15–31.

[1-s] Prestemon, J.P., Holmes, T.P., 2000. Timber Price Dynamics Following a Natural Catastrophe. Am. J. Agr. Econ. 82(1),145–160.

[3-s] Prestemon, J.P., Holmes, T.P., 2004. Market Dynamics and Optimal Timber Salvage After a Natural Catastrophe. Forest Sci. 50(4), 495–511.

[11-s] Schou, E., Jacobsen, J., Kristensen, K., 2012. An economic evaluation of strategies for transforming even-aged into near-natural forestry in a conifer-dominated forest in Denmark. For. Policy Econ. 20, 89–98.

[12-s] Straka, T.J., Baker, J.B., 1991. A Financial Assessment of Capital-Extensive Management Alternatives for Storm-Damaged Timber. South. J. Appl. For. 15(4), 208–212.

[9-s] Straka, T.J., Marsinko, A.P., Baumann, J.L., Haight, R.G., 1995. Site Preparation and Tree Planting Costs on Hurricane-Damaged Lands in South Carolina. South. J. Appl. For. 19(3), 131–138.

[8-s] Thorsen, B.J., Helles, F., 1998. Optimal stand management with endogenous risk of sudden destruction. Forest Ecol. Manag. 108(3), 287–299.

[2-s] Yin, R., Newman, D.H., 1999. An intervention analysis of Hurricane Hugo’s effect on South Carolina’s stumpage prices. Can. J. Forest Res. 29(6), 779–787.

**Wildlife damage** n_4_=5.

[2-wd] Clasen, C., Griess, V.C., Knoke, T., 2011. Financial consequences of losing admixed tree species: A new approach to value increased financial risks by ungulate browsing. For. Policy Econ. 13(6), 503–511.

[1-wd] Horne, P., Petäjistö, L., 2003. Preferences for Alternative Moose Management Regimes among Finnish Landowners: A Choice Experiment Approach. Land Econ. 79(4), 472–482.

[3-wd] Wam, H.K., Hofstad, O., 2007. Taking timber browsing damage into account: A density dependant matrix model for the optimal harvest of moose in Scandinavia. Ecol. Econ. 62(1), 45–55.

[4-wd] Ward,A.I., White, P.C.L., Smith, A., Critchley, C.H., 2004. Modelling the cost of roe deer browsing damage to forestry. Forest Ecol. Manag. 191(1–3), 301–310.

[5-wd] Weigand, J.F., Haynes, R.W., Tiedemann, A.R., Riggs, R.A., Quigley, T.M., 1993. Economic assessment of ungulate herbivory in commercial forests of eastern Oregon and Washington, USA. Forest Ecol. Manag. 61(1–2), 137–155.

**Ice, snow** n_5_=4.

[3-is] Goodnow, R., Sullivan, J., Amacher, G.S., 2008. Ice damage and forest stand management. J. Forest Econ. 14(4), 268–288.

[1-is] Kuboyama, H., Amano, M., Oka, H., 1997. Suitability of Long Rotations in Snow Risk Areas: A Stochastic Simulation of Timber Plantations. J. Forest Plan. 3(2), 99–106.

[4-is] Olschewski, R., Bebi, P., Teich, M., Wissen Hayek, U., Grêt-Regamey, A., 2012. Avalanche protection by forests — A choice experiment in the Swiss Alps. For. Policy Econ. 15, 19–24.

[2-is] Teich, M., Bebi, P., 2009. Evaluating the benefit of avalanche protection forest with GISbased risk analyses - A case study in Switzerland. Forest Ecol. Manag. 257(9),1910–1919.

**Multiple hazard** n_7_=24.

[9-mh] Bright, G., Price, C., 2000. Valuing forest land under hazards to crop survival. Forestry. 73(4), 361–370.

[21-mh] Broido, A., McConnen, R.J., O’Regan, W.G., 1965. Some Operations Research Applications in the Conservation of Wildland Resources. Manage. Sci. 11(9), 802–814.

[22-mh] Brown, C.G., Kellogg, L.D., 1996. Harvesting economics and wood fiber utilization in a fuels reduction project: a case study in eastern Oregon. Forest Prod. J. 46(9), 45-52.

[8-mh] Caulfield, J.P., 1987. Decision analysis in damaged forest plantations. Forest Ecol. Manag. 22(1–2), 155–165.

[13-mh] Dai, Y., Chang, H.H., Liu, W., 2014. Do forest producers benefit from the forest disaster insurance program? Empirical evidence in Fujian Province of China. For. Policy Econ. 50, 127-133.

[20-mh] DeWalle, D.R., Buda, A.R., Fisher, A., 2003. Extreme Weather and Forest Management in the Mid-Atlantic Region of the United States. North. J. Appl. For. 20(2), 61–70.

[4-mh] Dieter, M., 2001. Land expectation values for spruce and beech calculated with Monte Carlo modelling techniques. For. Policy Econ. 2(2), 157–166.

[11-mh] Knoke, T., 2008. Mixed forests and finance — Methodological approaches. Ecol. Econ. 65(3), 590–601.

[12-mh] Knoke, T., Stimm, B., Ammer, C., Moog, M., 2005. Mixed forests reconsidered: A forest economics contribution on an ecological concept. Forest Ecol. Manag. 213(1–3), 102–116.

[23-mh] Knoke, T., Wurm, J., 2006. Mixed forests and a flexible harvest policy: a problem for conventional risk analysis? Eur. J. For. Res. 125, 303-315.

[6-mh] Kuboyama, H., Oka, H., 2000. Climate Risks and Age-related Damage Probabilities – Effects on the Economically Optimal Rotation Length for Forest Stand Management in Japan. Silva Fenn. 34(2), 155–166.

[5-mh] Holecy, J., Hanewinkel, M., 2006. A forest management risk insurance model and its application to coniferous stands in southwest Germany. For. Policy Econ. 8(2),161–174. (+ Corrigendum For. Policy Econ. 38, 229).

[18-mh] Holmes, T.P., Aukema, J.E., Von Holle, B., Liebhold, A., Sills, E., 2009. Economic Impacts of Invasive Species in Forests: Past, Present, and Future. Ann. NY. Acad. Sci. 1162(1), 18–38.

[14-mh] Lewandrowski, J., Kim, C.S., Aillery, M., 2014. Carbon sequestration through afforestation under uncertainty. For. Policy Econ. 38, 90–96.

[7-mh] Lin, C.R., Buongiorno, J., 1998. Tree Diversity, Landscape Diversity, and Economics of Maple-Birch Forests: Implications of Markovian Models. Manage. Sci. 44(10), 1351–1366.

[15-mh] Loisel, P., 2011. Faustmann rotation and population dynamics in the presence of a risk of destructive events. J. Forest Econ. 17(3), 235–247.

[2-mh] Lovejoy, P.S., 1916. The Costs and Values of Forest Protection. J. Forest. 14(1), 24–38.

[16-mh] Notaro, S., Paletto, A., 2012. The economic valuation of natural hazards in mountain forests: An approach based on the replacement cost method. J. Forest Econ. 18(4), 318–328.

[17-mh] Notaro, S., Paletto, A., Raffaelli, R., 2009. Economic Impact of Forest Damage in an Alpine Environment. Acta silv. lignaria Hung. 5, 131–143.

[10-mh] Roessiger, J., Griess, V.C., Härtl, F., Clasen, C., Knoke, T., 2013. How economic performance of a stand increases due to decreased failure risk associated with the admixing of species. Ecol. Model. 255, 58–69.

[24-mh] Routledge, R.D., 1980. The effect of potential catastrophic mortality and Other Unpredictable events on optimal forest rotation policy. Forest Sci. 26(3), 389-399.

[19-mh] Stainback, G.A., Lavalapati, J.R.R., 2004. Modeling catastrophic risk in economic analysis of forest carbon sequestration. Nat. Resour. Model. 17(3), 299–317.

[3-mh] Staupendahl, K., Möhring, B., 2011. Integrating natural risks into silvicultural decision models: A survival function approach. For. Policy Econ. 13(6), 496–502.

[1-mh] Yin, R., Newman, D.H., 1996. The Effect of Catastrophic Risk on Forest Investment Decisions. J. Environ. Econ. Manag. 31(2), 186–197.

1. Bracketed texts refer to articles references in the database. [↑](#footnote-ref-1)
